# Monitoring Exercise-Induced Muscle Fatigue and Adaptations: Making Sense of Popular or Emerging Indices and Biomarkers

**DOI:** 10.3390/sports6040153

**Published:** 2018-11-26

**Authors:** George Theofilidis, Gregory C. Bogdanis, Yiannis Koutedakis, Christina Karatzaferi

**Affiliations:** 1 Muscle Physiology and Mechanics Group, School of Physical Education and Sports Science, University of Thessaly, Trikala 42100, Greece; gtheofilidis@uth.gr; 2 School of Physical Education and Sports Science, National and Kapodistrian University of Athens, Dafne 17237, Greece; gbogdanis@phed.uoa.gr; 3 Human Performance Laboratory, School of Physical Education and Sports Science, University of Thessaly, Trikala 42100, Greece; y.koutedakis@uth.gr; 4 Faculty of Arts, University of Wolverhampton, Walshall WS1 3BD, UK; 5 Experimental Myology & Integrative Physiology Cluster, FSHW, Plymouth Marjon University, Plymouth PL6 8BH, UK

**Keywords:** training adaptations, exercise induced muscle fatigue, fatigue index, fatigue agents, lactate monitoring, redox markers, muscle inflammation, oxidative stress monitoring

## Abstract

Regular exercise with the appropriate intensity and duration may improve an athlete’s physical capacities by targeting different performance determinants across the endurance–strength spectrum aiming to delay fatigue. The mechanisms of muscle fatigue depend on exercise intensity and duration and may range from substrate depletion to acidosis and product inhibition of adenosinetriphosphatase (ATPase) and glycolysis. Fatigue mechanisms have been studied in isolated muscles; single muscle fibers (intact or skinned) or at the level of filamentous or isolated motor proteins; with each approach contributing to our understanding of the fatigue phenomenon. In vivo methods for monitoring fatigue include the assessment of various functional indices supported by the use of biochemical markers including blood lactate levels and more recently redox markers. Blood lactate measurements; as an accompaniment of functional assessment; are extensively used for estimating the contribution of the anaerobic metabolism to energy expenditure and to help interpret an athlete’s resistance to fatigue during high intensity exercise. Monitoring of redox indices is gaining popularity in the applied sports performance setting; as oxidative stress is not only a fatigue agent which may play a role in the pathophysiology of overtraining syndrome; but also constitutes an important signaling pathway for training adaptations; thus reflecting training status. Careful planning of sampling and interpretation of blood biomarkers should be applied; especially given that their levels can fluctuate according to an athlete’s lifestyle and training histories.

## 1. Introduction

Skeletal muscle is a highly plastic tissue which adapts its morphology and metabolism according to external stimuli. One of the main functions of skeletal muscle is to convert chemical energy into mechanical work in order to support movement of the human body [1]. During muscle contraction, aerobic and anaerobic metabolic pathways contribute to energy supply according to the duration and intensity of muscle effort [2]. There is an inverse relationship between the duration and intensity of muscle effort, i.e., very intense muscle contractions can be maintained only for a short duration, while less intense contractions can be sustained or repeated for longer periods of time. The ability of an athlete to perform physical work is intrinsically linked to the metabolic pathways sustaining ATP requirements for the given muscular performance and may thus be delineated as: (a) the capacity to perform prolonged exercise, (b) the capacity to exercise at high-intensity for a relatively short time period, (c) the capacity to contract as fast as possible, and (d) generate maximal force instantaneously (explosive force/power generation) (for a discussion see [3]). These capacities are not developed to the same degree and can have a strong genetic background (e.g., see [4] on genetic determinants of exercise performance). Regular exercise with the appropriate intensity and duration may improve these capacities by modifying energy supply and enzymatic activities, as well as influencing muscle morphology and contractile function, resulting in improved performance during a given task, and an enhanced resistance to fatigue [5,6].

Both muscle acidosis [7,8] and reactive oxygen species [9] have been considered as factors which modulate muscle performance. Recent literature in the field of muscle fatigue indicates previously unappreciated interactions of fatigue factors as well as effects beyond an acute effect on the actomyosin interaction per se, which could contribute to a reduced power output [10,11,12]. Additionally, the different fiber types have variable resistance to fatigue [13].

The aim of this review is to discuss those aspects of the fatigue phenomenon that should inform the choice of monitoring indices and their interpretation. Because, there is plethora of functional indices [14,15] and biomarkers [16] of fatigue we are focusing on the interpretation of lactate, and oxidative stress indices namely total antioxidant capacity (TAC), protein carbonyls (PC) and thiobarbituric acid-reactive substances (TBARS), which are commonly used in the laboratory and field settings. It is hoped that our approach would be helpful for the professional who is active in the applied sports setting.

## 2. Materials and Methods

An online search of journal databases PubMed and Scopus was performed. The following keywords were used as search terms in various combinations: athletic/human performance, training adaptations, muscle fatigue, fatigue index, fatigue agents, lactate, acidosis, redox status, oxidative stress, total antioxidant capacity (TAC), protein carbonyls (PC), thiobarbituric acid reactive substances (TBARS). Articles and articles cited in the reference lists of identified journals were selected based on their relevance and specificity.

## 3. Skeletal Muscle Fatigue

Skeletal muscle fatigue has been generally defined as “the decrease in force or power production in response to contractile activity” [17]. However, more comprehensive definitions regarding exercise induced muscle fatigue have been introduced focusing either at the reversibility of loss of muscle force during exercise as discussed in [18] or in the witnessed deviation from the maximal or expected force or power that muscles can produce after the onset of the sustained exercise [19]. When studying skeletal muscle fatigue, muscle activation, vascular function, bioenergetics, changes in intracellular signaling and molecular mechanics should all be considered [17].

Muscle activation begins in the cortex, continues with excitation of lower motor neurons in the spinal cord, to the axon of the lower motor neuron and eventually to the neuromuscular junction of the muscle [20]. In this process, fatigue can potentially arise at any point of the pathway. When focusing on the processes inside the spinal cord and the brain, fatigue is defined as “central”, and when focusing on the peripheral nerve, neuromuscular junction, and the muscle, fatigue is defined as “peripheral” [21]. The nature of muscle fatigue depends on the characteristics of exercise, i.e., its intensity and duration, e.g., fatigue during a marathon run is different from fatigue during a series of repeated sprints [16]. However, although fatigue is evidenced by impaired force or power generation by the contractile proteins, it should be stressed that it is not caused by a single factor [18,19] and that various mechanisms are involved, each having a contribution that is specific to the task being performed, and the overall health status of the individual [19,22]. Studies on muscle fatigue have been conducted on humans, isolated muscles, isolated intact muscle fibers (for a review see [21]), isolated skinned muscle fibers (e.g., [10]) or even at the level of filamentous proteins [23], or isolated motor proteins (e.g., [24]) with advantages and disadvantages in each approach.

In vitro studies have shown that the impairment of muscle contraction, and thus the development of muscle fatigue at the cellular level, derives from either (a) alterations in excitability of the muscle fiber, (b) accumulation of metabolic by-products, (c) production of reactive oxygen species and (d) Ca^2+^ movements in the fiber compartments [10,21,23,25,26,27,28]. All of the above can be grouped in two major mechanisms that are responsible for the inhibition of muscle function witnessed during fatigue: (a) impairment at the level of activation, and b) impairment of the actin–myosin interaction [7].

In humans, fatigue manifests as an inability to continue a motor task at the required intensity, eventually leading to exhaustion. This performance decline is often called fatigability [14]. Methods for quantifying fatigue include measurements of the drop in peak force, torque or power of muscle contraction, expressed as a “fatigue index”, i.e., the percentage or rate of performance decrease over time [14]. That fatigue index may be taken as a measure of resistance to fatigue, and may be assessed using various ergometers. On an isokinetic dynamometer, fatigue resistance may be assessed (i) by the number of maximum effort repetitions until exhaustion or (ii) by the number of maximum effort repetitions until a 50% reduction in torque output is reached, or (iii) by the percent decline in torque from the beginning to the end of a predetermined time period [29]. Fatigue index may also be assessed using maximal sprint cycling tests, such as the Wingate test, by calculating the difference between the highest and lowest power output, expressed as a percentage of the highest power [30]. Also fatigue can be assessed by the drop of sprint performance during a repeated sprint test [31]. Of course such approaches disregard the temporal development of the drop in power output (i.e., is it steep thus ‘early’ or gradual) in the name of simplicity. For this reason, alternatives have been sought by us and others (see e.g., [32]) considering the whole time course of an exercise bout.

Other fatigue resistance assessment methods include measurement of the number of repetitions against a submaximal load during resistance exercise [33,34], or measurement of time to exhaustion during steady or varying pace submaximal or maximal intensity running or cycling [35,36]. At submaximal intensities, the ability to resist fatigue has been linked with substrate availability and especially glycogen in slow twitch muscle fibers [37], muscle activation [38], muscle fiber type [39], mitochondrial and oxidative enzymes activity [40] and capillary density [33].

At high or maximal intensities of exercise, fatigue resistance has been linked with fiber type composition, with individuals having high percentage of slow muscle fibers exhibiting less fatigue [41], and with average performance depending both on anaerobic and aerobic metabolism, whose contributions vary according to the test duration and intensity [42,43]. Also, energy availability at the required rates by anaerobic glycolysis and phosphocreatine breakdown have been linked with fatigue resistance [44,45,46,47,48,49].

It is clear from the above that ‘fatigue measurements’ are task-specific. One should consider carefully how one defines fatigue from a functional point of view for their given population and type of activity. As various biomarkers, in the blood, or infrequently in muscle, can provide metabolic information of interest to assist in the interpretation of the mechanisms underlying fatigue or training adaptation towards resisting fatigue, one needs to then consider carefully the use and interpretation of popular biomarkers.

## 4. Lactate as a Fatigue Agent and as a Signaling Molecule

**Figure 1:**
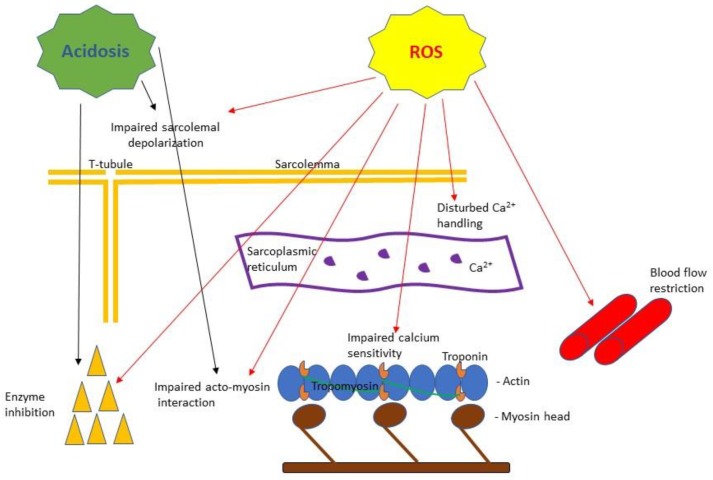
A simplified scheme of the purported effects of acidosis and reactive oxygen species (ROS) based on muscle fatigue literature reviewed in this article. Acidosis has been shown to cause impaired Ca^2+^ handling [55] and inhibition of key metabolic enzyme activities [57] as well as to inhibit myosin ATPase [56] affecting force and velocity of contraction [10]. ROS have been shown to cause: impaired sarcolemma ability to depolarize [87], disturbance in calcium release from the sarcoplasmic reticulum and decreased calcium sensitivity of the myofilaments [89], impaired acto-myosin interaction [91], enzyme inhibition [92] and blood flow restriction [93].

Lactic acid is the product of the anaerobic breakdown of carbohydrates and quickly dissociates to lactate and protons (hydrogen ions) promoting acidosis. Lactate accumulation was thought to indicate inadequate oxygen supply in the working muscles, although oxygen delivery is not always the main cause of lactic acid production [50,51,52]. The lactic acid hypothesis for muscle fatigue states that “accumulation of lactate or acidosis in working muscle causes inhibition of contractile processes, either directly or via metabolism, resulting in diminished exercise performance” [53]. Indeed, a strong connection between pH regulation and work capacity has been shown, suggesting that acidosis strongly contributes to fatigue [54]. More specifically, acidosis may impair function of contractile properties by reducing: (a) sarcoplasmic Ca^2+^ release and re-uptake, (b) myofibrillar Ca^2+^ sensitivity [55] and (c) activity of ATPase [56] and key enzymes of glycolysis such as phosphofructokinase and phosphorylase [57] (Figure 1). However, skinned fiber experiments in variable pH and temperatures have indicated that acidosis, while still important, is not the only reason behind the slowing of contractile velocity observed during fatigue (e.g., [10]). Human studies involving repeated sprint exercise, have shown that peak power output has shown that peak power output during a subsequent sprint is not affected by acidosis but is dependent on phosphocreatine availability [45,46,48,58]. Regarding lactate per se, a series of experiments conducted mostly on skinned muscle fibers bathed in lactate solutions with concentrations ranging from 15 to 40 mM while keeping a constant pH of 7.1, showed that the presence of lactate does not inhibit excitation contraction coupling [59,60]. Kristensen et al. 2005 [61], reported a small beneficial effect of lactate in restoring membrane depolarization caused by high external K^+^. Thus the intracellular accumulation of lactate per se is not a major factor in muscle fatigue.

**Figure 2:**
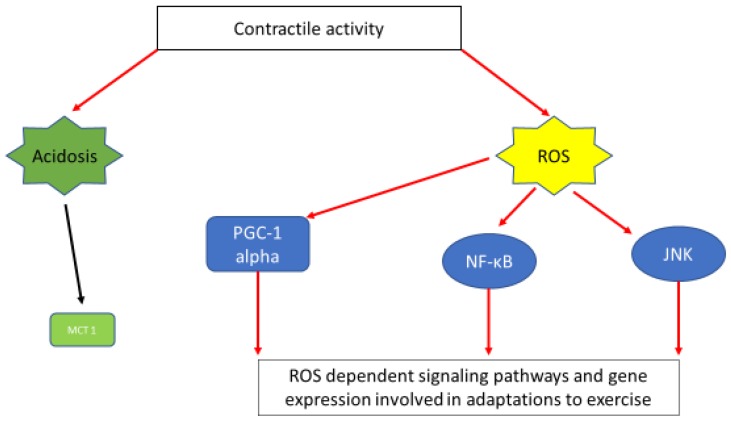
A simplified scheme depicting muscle contraction linked signaling pathways and gene expression induced via acidosis and ROS generation. Exercise-induced acidosis has been linked with induction of MCT1 expression [62]. Activation of important signaling pathways and gene expression connected with exercise adaptations, namely; PGC1a [99], NF-κΒ [97], JNK [98] are ROS dependent.

Besides their potential roles in fatigue, lactate and hydrogen ions in skeletal muscles can act as intracellular messengers that regulate physiological adaptations by promoting a number of transcription factors. For example, intracellular lactate is a signaling molecule for inducing monocarboxylate transporter 1 (MCT1) expression [62] (Figure 2). Furthermore, lactate has been shown to promote aerobic adaptations by inducing intramuscular triglyceride (TG) accumulation and mitochondrial maintenance in mouse myotubes (C2C12 cell line) [63], or by regulating intramuscular triglyceride metabolism via transforming growth factor-b1 mediated pathways during post-exercise recovery from strenuous exercise in rats [64].

Due to the invasive nature of lactate measurements in the muscle, blood lactate measurements are used as a practical method of estimating acid–base status in the muscle. This assumes that blood lactate reflects muscle lactate, although this is not always the case, especially during intense and intermittent exercise, where lactate efflux from the working muscle and lactate distribution to other muscles or tissues is unbalanced [65]. Typically lactate levels are monitored in the blood before and after the performance of all-out intense exercise like the Wingate 30-s test and other shorter sprint tests [66], or before, during and at the end of graded exercise protocols [67]. Levels of 18.8 ± 1.6 mmol/L have been measured in trained sprinters [68], while team sport players record levels as high as 13.3 ± 1.9 mmol/L [69] or as low as (7.8 ± 1.6) depending on position played [69], training status [70], and gender [71].

Change of blood lactate concentration during graded exercise (Lactate Curve) sets multiple threshold concepts and training intensity domains, while a right shifted curve following training is indicative of improvement in aerobic fitness [72]. A popular concept is the ‘lactate threshold’ which is defined as the exercise intensity at which a certain blood lactate concentration is attained, e.g., 4 mmol/L [73]. Lactate threshold is considered to be linked with the fatigue process, since above this exercise intensity, blood lactate is rapidly accumulated and this may reflect increases in muscle acidity and rapid glycogen depletion via anaerobic glycolysis [72].

However, interpretations of such thresholds and relative exercise intensity domains have to be made on solid testing procedures as recent research highlighted the diversity of such outcomes after manipulating graded test variables [74]. Additionally, other factors, such as nutrition before the test are important when blood lactate is used for monitoring purposes. For example, prior exercise that depleted glycogen levels [75] or a high carbohydrate diet [76] can influence blood lactate concentration and the timing of the achievement of the predefined thresholds.

## 5. Exercise-Induced Oxidative Stress and Inflammation

Oxidative stress, originally defined as “a disturbance in the pro-oxidant– antioxidant balance in favor of the former”, has acquired more comprehensive definitions such as “an imbalance between oxidants and antioxidants in favor of the oxidants, leading to a disruption of redox signaling and control and/or molecular damage” [77]. This balance between oxidants and antioxidants is also termed as redox balance or redox homeostasis [78]. Exercise induces oxidative stress by producing free radicals and other reactive oxygen species (ROS) which may transiently overcome the antioxidant capacity. A free radical is defined as any molecular species that contains at least one unpaired electron; chemically, they are highly reactive because the unpaired electron attempts to stabilize itself by pairing with another electron [79]. Because of their chemical instability, free radicals are capable of inflicting biological damage [80]. The main free radicals formed in cells are superoxide (^•^O^−^_2_) and nitric oxide (NO) [77]. ROS is a general term that refers not only to oxygen-centered radicals but also includes non-radical but reactive oxygen derivatives (e.g., hydrogen peroxide) and the term reactive nitrogen species (RNS) refers to both nitrogen radicals as well as other reactive molecules where the reactive center is nitrogen [81]. Redox homeostasis can be studied in blood or in muscle tissue with detection and measurements of certain biomarkers that fall into one of the following categories: (1) oxidants (e.g., superoxide anions), (2) enzymatic (e.g., catalase—CAT) or nonenzymatic antioxidants (e.g., uric acid, total antioxidant capacity—TAC), (3) oxidation products (e.g., protein carbonyls), (4) antioxidant/pro-oxidant balance (e.g., GSH/GSSG ratio) [82].

Exercise has been shown to cause redox disruption through many different mechanisms. The most prominent exercise induced oxidative stress mechanisms include: (1) the mitochondrial electron transfer system within muscle cells, (2) ROS production via Xanthine oxidase within endothelial cells, (3) ROS production via nicotinamide adenine dinucleotide phosphate oxidase (NADPH) [77], (4) infiltrating phagocytes attacking degenerated cells deriving from exercise induced muscle damage [83], (4) Reduction in blood flow toward splanchnic organs during exercise (under-perfusion) and subsequent post exercise re-sustained blood flow (reperfusion) [84,85].

The latter indicates that attention should be paid, when taking an athlete’s history, in recording recent activity but also overall training history involving exposure to eccentric loads. Unaccustomed or novice, exercise testing participants may exhibit higher levels of inflammatory and/or redox markers; all participants should avoid such exercise testing if close to undertaking clinical biochemistry assessments (e.g., yearly check-up) as the testing activity may confound their readings.

## 6. Reactive Oxygen Species (ROS) as Fatigue Agents and Signaling Molecules

Evidence supporting the role of ROS as agents causing fatigue has come from either laboratory muscle preparations at conditions mimicking fatigue (direct exposure to ROS) or from human studies using antioxidants as a pretreatment to decrease fatigue [86]. Laboratory experiments on muscle fibers have shown that ROS can cause a direct inhibition of force production (Figure 1) by: (1) compromising sarcolemma ability to depolarize [87], (2) causing disturbance in calcium handling from the sarcoplasmic reticulum [88,89], (3) causing a decrease in the calcium sensitivity of the myofilaments [89,90], and (4) having a direct effect on acto-myosin interaction [91]. Furthermore, ROS can indirectly inhibit force production by disrupting muscle ability to sustain a certain level of force production by suppressing bioenergetics availability (i.e., reduced lipid oxidation by mitochondria due to inhibition of Carnitine Palmitoyltransferase I (CPT I) activity, or limited blood supply [92,93]).

Nevertheless, it has to be noted that although high levels of reactive oxygen species may result in contractile dysfunction and fatigue, physiological levels of reactive oxygen species are required for normal force production in unfatigued skeletal muscle [82,94]. There appears to be an optimal redox balance for efficient force generation at the cross-bridge level as indicated by skinned fiber work (e.g., [11]). Additionally, in muscle preparations of animals with elevated levels of antioxidants administered by diet [94], or treated with ROS/RNS-neutralizing compounds [95], or transgenic overexpression [96], there was no evident improvement of fatigability or recovery from fatigue. Thus, maximizing availability of antioxidants is not a straightforward avenue for improving fatigue resistance.

Exercise-induced ROS also act as intracellular messengers that regulate exercise induced adaptations with respect to muscle hypertrophy and oxidative metabolism [9]. More specifically, the administration of a xanthine oxidase inhibitor (allopurinol), reduced NF-κB activation in response to sprinting exercise in rats, [97]. In humans, the exercise-induced increase in JNK phosphorylation, was blocked by infusion of the ROS scavenger N-acetylcysteine (NAC), while analysis of ROS-sensitive genes demonstrated a ROS dependent exercise-induced mRNA expression of the antioxidant enzyme manganese superoxide dismutase (MnSOD), suggesting that inhibition of ROS attenuates some skeletal muscle cell signaling pathways and gene expression involved in adaptations to exercise [98]. Furthermore, it has been shown that in the skeletal muscle of rats, peroxisome proliferator-activated γ receptor coactivator (PGC-1a) signaling pathways are redox sensitive and that non-mitochondrial ROS play an important role in stimulating mitochondrial biogenesis [99]. Finally, in a recent in vivo human study and in the absence of any exogenous redox manipulation, the vital role of reactive oxygen and nitrogen species produced during exercise in adaptations was substantiated for the first time [100].

A commonly used marker of antioxidant status is total antioxidant capacity (TAC), while protein carbonyls (PC) and thiobarbituric acid reactive substances (TBARS) are markers of protein oxidation and lipid peroxidation respectively. Although lipid peroxidation has been suggested as a potential fatigue marker after intense or exhaustive exercise [101], recent studies have criticized TBARS assay and, therefore care should be taken when interpreting changes in TBARS as an index of lipid peroxidation in vivo [102]. Periodic monitoring of those redox status indices (among others) is gaining importance for long term health evaluations, as increased oxidative stress seems to have a role in the pathophysiology of overtraining syndrome and impaired adaptation to exercise [103]. An increase in oxidative stress indices in athletes who participate in high-level training programs may reflect the existence of two or more stressors (high level of physical activity and severe infection) and frequent monitoring might allow such risk factors to be identified [104]. White blood cell (WBC) activity not only reflects the status of the immune system but is affected by muscle damaging exercise as well. Recently, monitoring the redox status of WBC, is emerging as an approach for long-term managing of elite athletes (to better prevent overtraining or treat an early infection [105]) and further innovations in this area are expected. Future research should focus in clarifying the links between the redox status of leukocyte subpopulations with performance, injury and training adaptations, as it has been shown that training status [106] and antioxidant supplementation [107] alters ROS production by WBC.

TAC: TAC is calculated against a pro-oxidant source, thus it is a non-specific estimation of the antioxidant capacity of a biological sample (for a discussion see [108]). Still, it is extensively used, and while it should be interpreted with caution [109] we consider that when some dietary precautions have been taken, its meaning improves. TAC is measured in mmol DPPH·L^−1^, and blood levels of 0.93 ± 0.08 mmol DPPH·L^−1^, are reported in healthy athletes at rest, with post exercise levels ranging between approximately +20% and −40% of resting values [110].

The magnitude of both acute post-exercise and few hours post-exercise TAC concentration has been shown to be affected by exercise intensity and modality (HIIT vs. continuous aerobic exercise) [111]. However, long-term training induced changes in TAC are questionable, since some studies show improvement of antioxidant defense system while others have shown no change or a decrease [109], and this discrepancy might reflect lifestyle habits and not training effects. Nevertheless, antioxidant capacity has been shown to reflect training load fluctuations [112,113] and this has to be taken into account when interpreting TAC in individuals who are regularly training and whose dietary and other lifestyle habits are not changing.

PC: Intense exercise causes acute increases in PC [111]. However, aerobic exercise with an eccentric component (downhill running) has also an effect on both acute and later in time post-exercise PC increases [114]. Blood levels of ~0.6 nmol·mg^−1^ protein have been reported in healthy individuals at rest and ~1.1 nmol·mg^−1^ protein (83% increase) 24 hours post-eccentric exercise [115]; and ~0.70 nmol·mg^−1^ protein post-graded exercise [116]. Limited evidence exists on intramuscular levels of PC with post-exercise blood levels adequately reflecting post-exercise muscle levels [117].

Carbonylation tags proteins for degradation (catabolism) or carbonylated proteins may form aggregates that can become cytotoxic; these have been associated with a large number of age-related disorders [118] and it is not yet known if they are formed in young and healthy muscle. In the context of sports practice, one could monitor muscle mass (absolute and relative) and use PC levels in order to flag up possible muscle loss.

TBARS: High levels of resting TBARS values in the blood have been related with lower aerobic capacity and impaired skeletal muscle energy metabolism in patients with metabolic syndrome [119] and sedentary women [120]. In athletes, although different long-term training strategies establish influence basal lipid peroxidation levels, TBARS level cannot be predicted by maximal oxygen uptake [121]. Although sodium bicarbonate resulted in lower post-exercise TBARS in the blood, when compared with a placebo group, it had no effect on a repeated sprint test performance, suggesting no relationship between blood TBARS and fatigue [122].

Overall, despite the fact that muscle redox status disturbances have been associated with muscle fatigue in vitro, a causal effect between fatigue resistance and oxidative stress indices has not yet been established in humans. This may be because of the extreme complexity of the in vivo redox biology and thus more sophisticated research on redox specificity in relation to muscle fatigue needs to be done [123]. Moreover, it can’t be assumed that blood redox indices reflect their intramuscular levels. In the majority of human studies redox status indices have been evaluated in blood and results were then extrapolated in tissues. Another issue emerging from the literature is that temporal considerations may be underestimated [124]. For the markers mentioned here, changes in their levels following muscle damage seem to peak at 48h with TBARS remaining elevated in 72 h as well [125]. 

Limited work has concurrently examined blood and skeletal muscle levels. Recent work from our group (Poulianiti, personal communication) on an animal model of disease has shown no clear correlation between resting blood and resting muscle levels of these indices. However, Veskoukis et al. (2009) [117] reported that some redox status indices in blood (including PC) adequately reflected the oxidative stress changes that happened in healthy rat gastrocnemius after exercise and/or xanthine oxidase inhibition. Rodriguez et al. (2012) [126] however found that only PC concentrations were moderately correlated between blood and skeletal muscle (vastus lateralis) levels in patients with chronic obstructive pulmonary disease.

It thus appears that blood PC levels may reflect the exercise effect on muscle’s carbonylation status and perhaps can help in the estimation of muscle’s redox status following exercise. More attention may be needed on the interplay between the exercise-induced redox response and the overall immune status of an athlete to assess their recovery from injury. Whichever marker one uses allowances might be necessary for repeated blood sampling over a period of time, or on a time point not immediately post-exercise, as various markers may peak at different time points, depending also on an athletes overall training, immune and nutritional status.

## 7. Conclusions

Exercise-induced muscle fatigue is a complex phenomenon, with several experimental approaches and categorizations with advantages and disadvantages in each approach. Regarding functional monitoring of muscle performance, one should consider the time course of fatigue development and be clear on defining fatigue based on testing parameters that are relevant to the sport activity. Acidosis as well as redox balance disruptions in working muscles may contribute to or reflect the reduction of muscle performance, especially in intense or sprint type exercise, and thus monitoring such related biomarkers can assist in the interpretation of the fatigue phenomenon.

Blood lactate measurements are used as a practical method of estimating acid–base status and metabolic contributions but solid testing procedures such as standardized testing protocols in terms of exercise intensity progression and stage duration, as well as controlled nutrition prior to testing are needed. Monitoring redox disturbances needs careful consideration, including immune and nutritional status, and poses some practical challenges as different factors may peak at different time points. A new direction in monitoring the immune status via the redox assessment of WBC may hold promise in preventing overtraining.

Both lactate and protons and oxidative stress factors, also act as intracellular messengers that regulate physiological adaptations. Thus, apart from their use as markers of fatigue their role in certain domains of muscle function as in bioenergetics (e.g., mitochondrial respiration), muscle contraction (e.g., inhibition of actomyosin interaction), muscle damage (e.g., exercise induced muscle inflammation) and fatigue resistance need further investigation, to shed light to the interplay between cell signaling induced by these molecules and adaptations to a chronic exercise stimulus.
